# Injury and depression among 212 039 individuals in 40 low- and middle-income countries

**DOI:** 10.1017/S2045796019000210

**Published:** 2019-05-14

**Authors:** A. Stickley, H. Oh, T. Sumiyoshi, M. McKee, A. Koyanagi

**Affiliations:** 1 Department of Preventive Intervention for Psychiatric Disorders, National Institute of Mental Health, National Center of Neurology and Psychiatry, 4-1-1 Ogawahigashi, Kodaira, Tokyo 187-8553, Japan; 2 The Stockholm Center for Health and Social Change (SCOHOST), Södertörn University, Huddinge 141 89, Sweden; 3 University of Southern California, Suzanne Dworak Peck School of Social Work, 1149 South Hill Street Suite 1422, Los Angeles, CA 90015, USA; 4 Department of Public Health and Policy, London School of Hygiene and Tropical Medicine, London, UK; 5 Research and Development Unit, Parc Sanitari Sant Joan de Déu, CIBERSAM, Barcelona, Spain; 6 ICREA, Pg. Lluis Companys 23, Barcelona, Spain

**Keywords:** Depression, epidemiology, injury, meta-analysis, World Health Survey

## Abstract

**Aims:**

Although injuries have been linked to worse mental health, little is known about this association among the general population in low- and middle-income countries (LAMICs). This study examined the association between injuries and depression in 40 LAMICs that participated in the World Health Survey.

**Methods:**

Cross-sectional information was obtained from 212 039 community-based adults on the past 12-month experience of road traffic and other (non-traffic) injuries and depression, which was assessed using questions based on the World Mental Health Survey version of the Composite International Diagnostic Interview. Multivariable logistic regression analysis and meta-analysis were used to examine associations.

**Results:**

The overall prevalence (95% CI) of past 12-month traffic injury, other injury, and depression was 2.8% (2.6–3.0%), 4.8% (4.6–5.0%) and 7.4% (7.1–7.8%), respectively. The prevalence of traffic injuries [range 0.1% (Ethiopia) to 5.1% (Bangladesh)], and other (non-traffic) injuries [range 0.9% (Myanmar) to 12.1% (Kenya)] varied widely across countries. After adjusting for demographic variables, alcohol consumption and smoking, the pooled OR (95%CI) for depression among individuals experiencing traffic injury based on a meta-analysis was 1.72 (1.48–1.99), and 2.04 (1.85–2.24) for those with other injuries. There was little between-country heterogeneity in the association between either form of injury and depression, although for traffic injuries, significant heterogeneity was observed between groups by country-income level (*p* = 0.043) where the pooled association was strongest in upper middle-income countries (OR = 2.37) and weakest in low-income countries (OR = 1.46).

**Conclusions:**

Alerting health care providers in LAMICs to the increased risk of worse mental health among injury survivors and establishing effective trauma treatment systems to reduce the detrimental effects of injury should now be prioritised.

## Introduction

Injuries and mental illness are important contributors to the global burden of disease that have both been relatively neglected in global policy discourse. The 2016 Global Burden of Disease Study (GBD 2016 DALYs and HALE Collaborators, 2017) estimated that injuries accounted for 10.7% of total Disability-Adjusted Life Years (DALYs) lost, with transport injuries accounting for 30.6%, unintentional injuries 42.1% and intentional injuries 23.0% of this figure. Mental and substance use disorders accounted for 6.8% of total DALYs, with depressive disorders accounting for 27.2% of the total. Beyond the human toll, the economic costs are enormous. A recent World Health Organization (WHO) report estimated that fatal and non-fatal road traffic injuries may cost as much as 2% of gross domestic product (GDP) in high-income countries (HICs) and 5% in some low- and middle-income countries (LAMICs) (World Health Organization, 2014) while the global cost of mental illness has been estimated, in 2010, at US$ 2.5 trillion (Trautmann *et al*., 2016).

Despite the huge social and economic costs of injuries and depression, a low priority has been given to both of these conditions in LAMICs. This is alarming for several reasons. First, over 90% of injury-related deaths occur in these countries, leading them to be described as a ‘neglected burden’ (Gosselin *et al*., 2009). Second, although an earlier study reported that injuries accounted for a similar share of the total DALYs in both HICs and LAMICs, it also noted that trends seem to be diverging (Higashi *et al*., 2015) with DALYs from road traffic injuries declining in HICs but increasing in low-income countries (LICs) (GBD 2016 DALYs and HALE Collaborators, 2017). Third, LAMICs also bear a much greater share of the burden of depressive disorders, with more than 80% of the global total of Years Lived with Disability (World Health Organization, 2017), yet these conditions typically receive <2% of the health budgets in these countries (Jacob *et al*., 2007).

Although there is increasing recognition that both mental illness and injuries are important health issues in developing countries (Nambiar *et al*., 2017), as yet, there has been little research on their association even though extensive research in HICs has highlighted the existence of a relationship, with different forms of injury being associated with an increased risk for poorer mental health outcomes such as depression (O'Donnell *et al*., 2004) and worse mental health occurring as a long-term consequence of injury (Mayou and Bryant, 2002). Moreover, depression has been linked to poorer post-injury outcomes including worse quality of life, an increased risk for disability/role-related disability and not returning to work (O'Donnell *et al*., 2005, 2009; Zatzick *et al*., 2008). Given the potential relationship between injury and depression and how it might place an even greater strain on population and societal well-being in LAMICs with fewer resources, research aimed at gaining a better understanding of the association between these conditions in LAMICs may have important public health implications. Importantly, two recent studies found that non-fatal traffic injuries and falls were respectively associated with treatment for depression and depression in LAMICs (Peltzer *et al*., 2015; Stewart Williams *et al*., 2015). However, these studies were restricted to older adults aged ⩾50 years and included just six countries.

In this study, we seek to further understanding of the injury-depression association with an analysis of predominantly nationally representative data from 40 LAMICs. This study thus has three aims: (1) to determine the prevalence of traffic and other (non-traffic) injuries among adults in the general population; (2) to examine the factors associated with injury; and (3) to explore whether traffic-related and other injuries are associated with an increased risk for depression among the general adult population, and to assess the extent to which any associations vary among countries.

## Methods

### The survey

The World Health Survey (WHS) was a cross-sectional, community-based study undertaken in 2002–2004 in 70 countries worldwide. Details of the survey are provided on the WHO website (http://www.who.int/healthinfo/survey/en/). Briefly, data were collected using stratified multi-stage random cluster sampling. Individuals aged ⩾18 years with a valid home address were eligible to participate. Each member of a selected household had an equal probability of being chosen using Kish tables. A standardised questionnaire, translated into the local language was used in all countries. The individual response rate across all countries was 98.5% (Nuevo *et al*., 2012). Approval to conduct the study was obtained from ethical committees at each study site. Informed consent was obtained from all participants. Sampling weights were generated to adjust for non-response and the population distribution reported by the United Nations Statistical Division.

Data were publicly available for 69 countries. Of these, ten countries were excluded from the present study due to a lack of sampling information. Ten HICs were excluded because our focus is on LAMICs. Then, eight LAMICs were deleted as >25% of the values for at least one of the variables used in the analysis were missing. Finally, Vietnam was also excluded as stable estimates could not be obtained due to the extremely low prevalence of depression. Thus, the final sample consisted of 40 LAMICs (*n* = 212 039). According to the World Bank classification at the time of the survey (2003), these included 17 LICs (*n* = 89 690), 14 lower middle-income countries (LMICs) (*n* = 65 184) and nine upper middle-income countries (UMICs) (*n* = 57 165). The data were nationally representative for all countries with the exception of China, Comoros, Ivory Coast, India and Russia. The included countries and their sample sizes are provided in the online Supplementary Material Table S1.

## Variables

### Depression

Depression was assessed using questions based on the World Mental Health Survey version of the Composite International Diagnostic Interview. Specifically, past 12-month depression related to the duration and persistence of depressive symptoms. Algorithms based on DSM-IV (American Psychiatric Association., 1994) used in previous WHS publications were employed (Cifuentes *et al*., 2008; Loerbroks *et al*., 2012). Respondents were first asked five questions. Those who answered ‘Yes’ to four of them were considered as possibly having depression or a major depressive episode. Specifically, respondents were asked: ‘During the last 12 months have you ever experienced…’: (a) A period lasting several days when you felt sad, empty or depressed? (b) A period lasting several days when you lost interest in most things you usually enjoy such as hobbies, personal relationships or work? (c) A period lasting several days when you have been feeling your energy level decreased or that you were tired all the time? (d) Did you lose your appetite? (e) Did you notice any slowing down in your thinking?’. Among those with possible depression, individuals who further responded ‘Yes’ to both of the following questions were classified as having depression: (a) Was this period for more than 2 weeks? (b) Was this period most of the day, nearly every day?

### Injury

Experience of a road traffic accident was assessed with the question ‘In the past 12 months, have you been involved in a road traffic accident where you suffered from bodily injury? This could have been an accident in which you were involved either as the occupant of a motor vehicle, or when you were riding a motorcycle or bicycle, or walking.’ Other (non-traffic) injuries were assessed with the question ‘In the past 12 months, have you suffered bodily injury that limited your everyday activities, due to a fall, burn, poisoning, submersion in water, or by a firearm, sharp weapon or an act of violence from another person?’ Both questions had ‘Yes’ and ‘No’ answer options. In the present study, both types of injury were combined in the category ‘Any injury’.

### Control variables

The control variables were selected based on past literature on factors associated with depression and/or injury (Clausen *et al*., 2015) and included sex, age, wealth, highest level of education achieved (< or ⩾ secondary: where secondary education or higher referred to secondary school completed, high school completed, college/pre-university/university completed and postgraduate degree completed), setting (rural or urban) and lifetime alcohol consumption which was assessed with a question that asked ‘Have you ever consumed a drink that contains alcohol (such as beer, wine, etc.)?’ with ‘Yes’ or ‘No’ answer options. All respondents who answered yes were classified as lifetime alcohol users. Cross-country wealth quintiles were created using principal component analysis based on 15–20 assets selected to be relevant to the country concerned. Information on smoking was obtained with a question which asked, ‘Do you currently smoke any tobacco products such as cigarettes, cigars, or pipes?’ Those who answered affirmatively were classified as current smokers.

### Statistical analysis

Statistical analyses were performed with Stata 14.1 (Stata Corp LP, College Station, Texas). The age- and sex-adjusted prevalence of traffic injury, other (non-traffic) injury, any injury, and depression was calculated using the United Nations population pyramids for the year 2010. The difference in sample characteristics by any injury was tested with Chi-squared tests. Multivariable logistic regression analysis was conducted to assess the association between injury (traffic injury, other injury, any injury) (exposure) and depression (outcome) across countries while adjusting for age, sex, wealth, education, setting (rural/urban), alcohol consumption and smoking. In order to assess between-country heterogeneity in the association between injury and depression, we calculated Higgins's *I*^2^ which represents the degree of heterogeneity that is not explained by sampling error with a value of 25% often considered as low, 50% as moderate and 75% as high heterogeneity (Higgins *et al*., 2003). A pooled estimate was obtained by combining the estimates for each country into a fixed effect meta-analysis (overall and by country-income level). Heterogeneity between groups by country-income level was tested with Cochran's Q tests.

All variables were included in the regression analysis as categorical variables with the exception of age (continuous variable). Taylor linearisation methods were used in all analyses to account for the sample weighting and complex study design. Results from the logistic regression analyses are presented as odds ratios (ORs) with 95% confidence intervals (CIs). The level of statistical significance was set at *p* < 0.05.

## Results

**Fig. 1.**
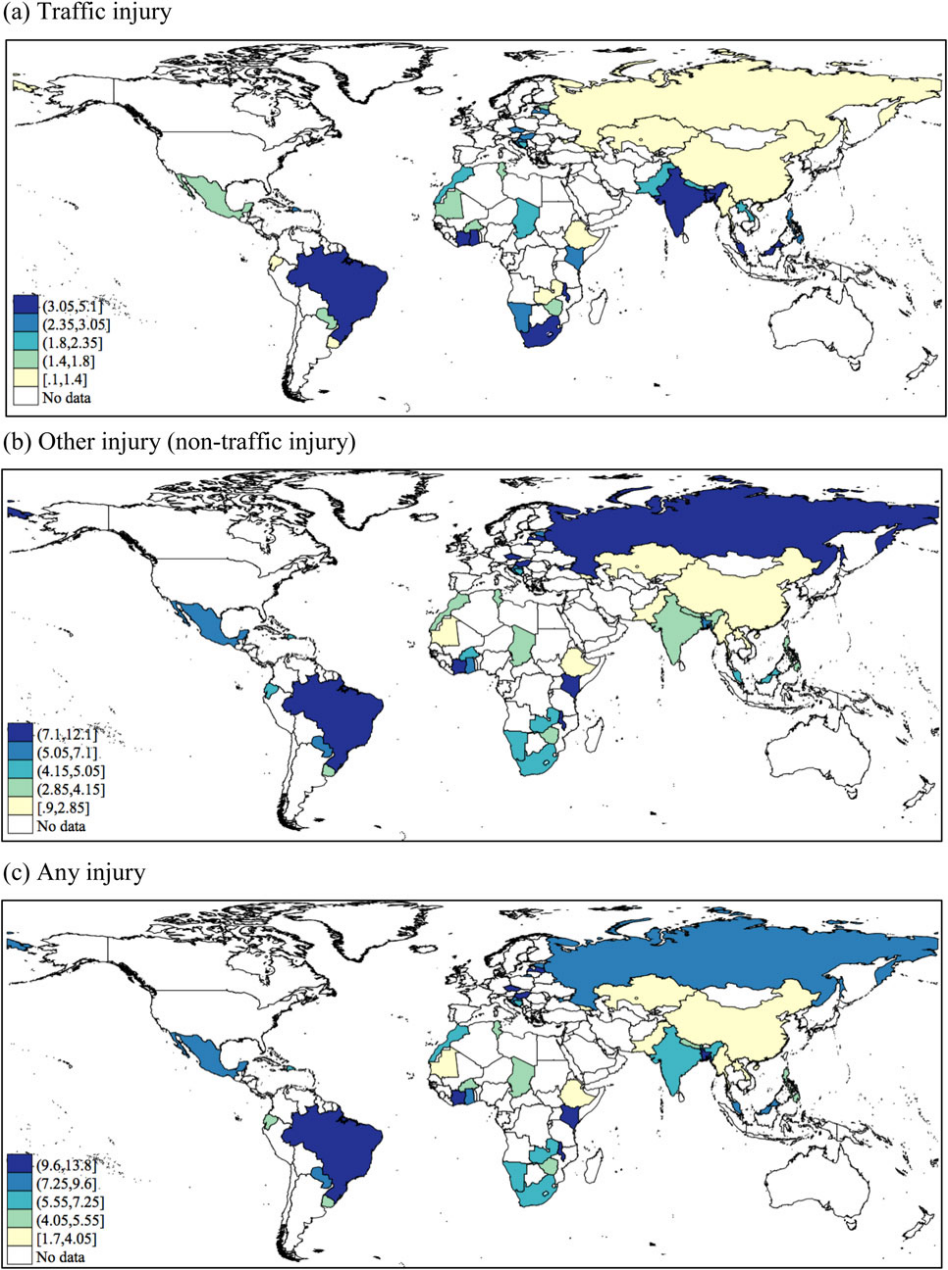
Age- and sex-adjusted past 12-month prevalence of (a) traffic injury, (b) other injury (non-traffic injury) and (c) any injury by country. All age-sex adjusted weighted estimates were calculated using the United Nations population pyramids for the year 2010.

**Fig. 2.**
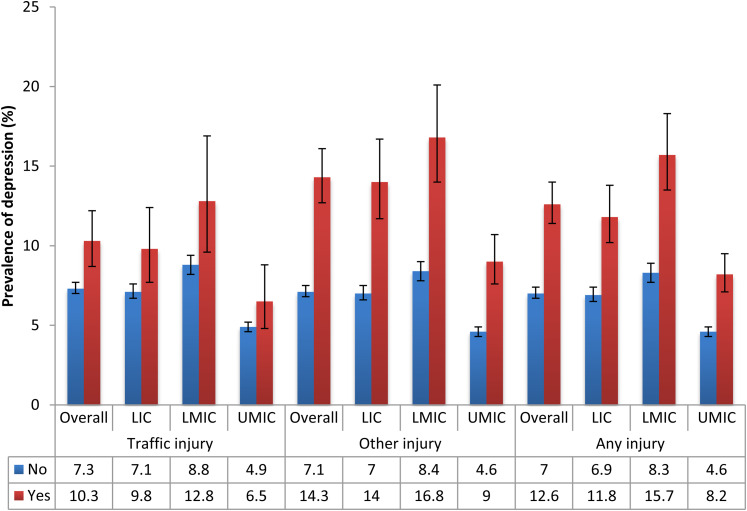
Prevalence of depression by the presence or absence of injury. LIC: Low-income countries, LMIC: Lower middle-income countries, UMIC: Upper middle-income countries. Bars denote 95% confidence intervals. All differences were statistically significant (*p* < 0.05) with the exception of traffic injuries in UMIC (*p* = 0.066).

The sample consisted of 212 039 individuals aged ⩾18 years [mean (standard deviation–s.d.) age 38.1 (15.9) years; 50.6% female]. The overall prevalence (95% CI) of past 12-month traffic injury, other injury, any injury and depression was 2.8% (2.6–3.0%), 4.8% (4.6–5.0%), 7.1% (6.8–7.4%) and 7.4% (7.1–7.8%), respectively. The prevalence of other injury and any injury was higher in LMICs and UMICs than in LICs [*χ*^2^ (2df) *p* < 0.0001] (online Supplementary Material Table S1). The prevalence of traffic injuries [range 0.1% (Ethiopia) to 5.1% (Bangladesh)], other injuries [range 0.9% (Myanmar) to 12.1% (Kenya)] and any injury [range 1.7% (Ethiopia, Myanmar) to 13.8% (Kenya)] varied widely between countries (online Supplementary Material Table S1; Fig. 1). Those who reported an injury were significantly more likely to be male, younger, better educated, live in an urban setting, consume alcohol and smoke (Table 1). The prevalence of depression was higher among those who experienced an injury compared to those who had not (e.g. 12.6% *v*. 7.0% for any injury in the overall sample) (Fig. 2).

**Table 1.** Sample characteristics (overall and by any injury)

			Any injury		
Characteristic	Category	Overall (%)	No (%)	Yes (%)	*p*-value^a^
Sex	Female	50.6	51.6	38.2	<0.0001
	Male	49.4	48.4	61.8	
Age (years)	18–34	48.6	48.3	52.7	<0.0001
	35–59	38.9	39.2	35.6	
	≥60	12.5	12.6	11.7	
Wealth	Poorest	20.1	20.2	19.5	0.3856
	Poorer	20.0	20.0	19.3	
	Middle	19.9	19.9	19.7	
	Richer	20.0	20.0	20.0	
	Richest	20.0	19.9	21.4	
Education	<Secondary	59.8	60.1	55.4	<0.0001
	≥Secondary	40.2	39.9	44.6	
Setting	Rural	56.2	56.4	49.8	<0.0001
	Urban	43.8	43.6	50.2	
Alcohol consumption	No	67.3	68.1	56.0	<0.0001
	Yes	32.7	31.9	44.0	
Smoking	No	73.3	74.0	63.8	<0.0001
	Yes	26.7	26.0	36.2	

^a^*p*-value was calculated with χ^2^ tests.

**Fig. 3.**
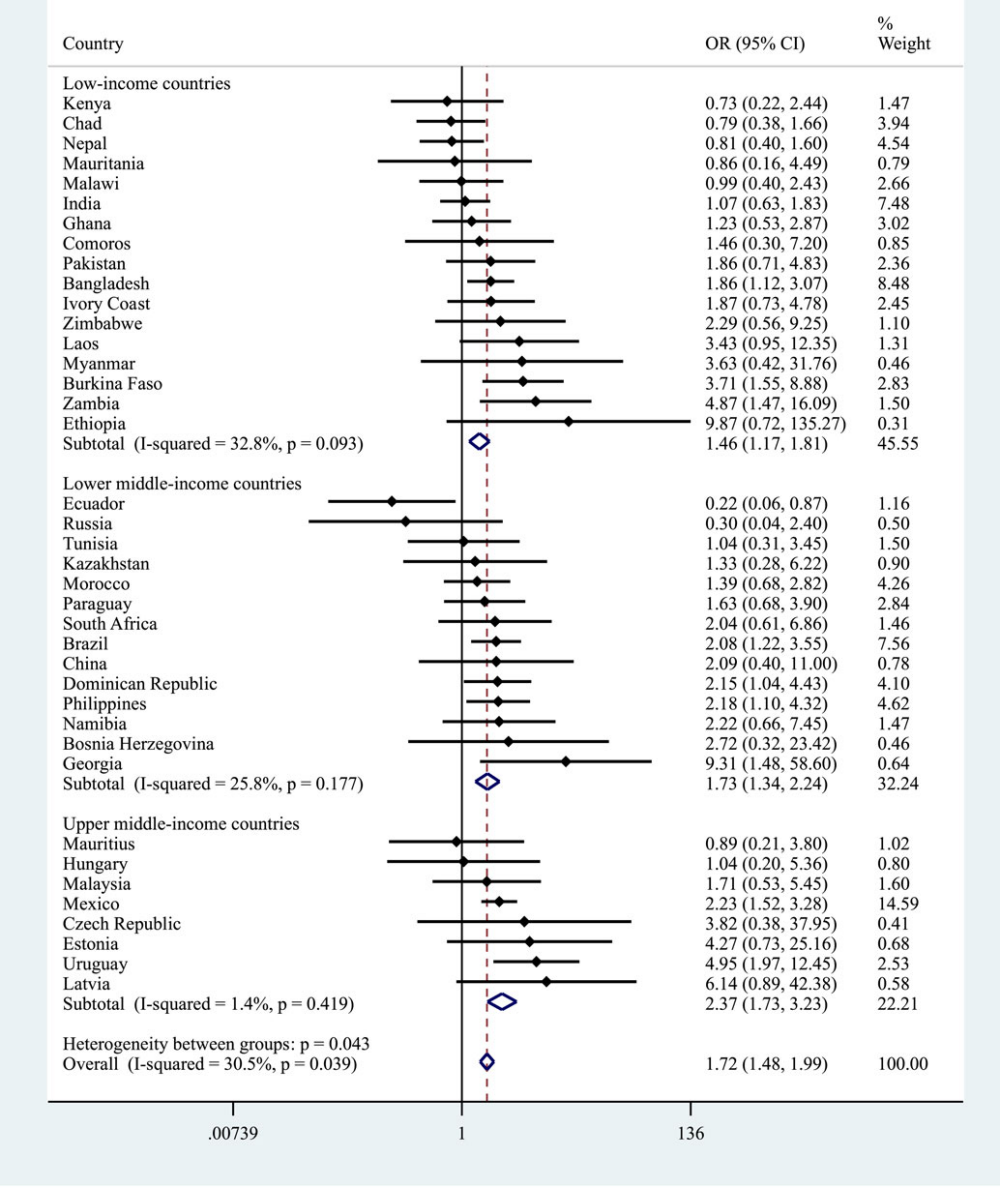
Country-wise association between traffic injury and depression estimated by multivariable logistic regression. OR, Odds ratio; CI, Confidence interval. Models were adjusted for age, sex, wealth, education, setting (rural/urban), alcohol consumption and smoking. Overall estimates were obtained by meta-analysis with fixed effects. Estimates for Croatia could not be obtained due to the small sample size.

**Fig. 4.**
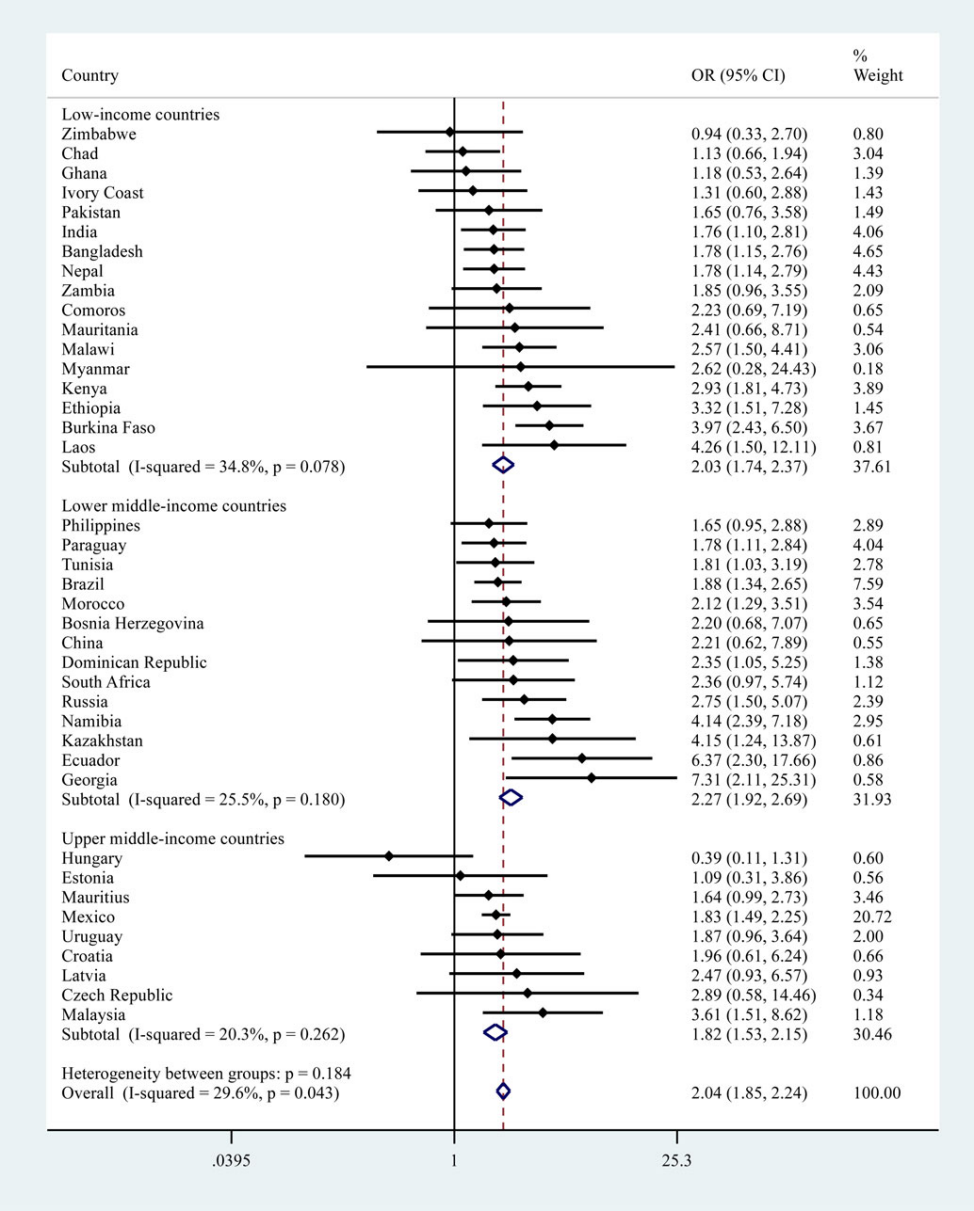
Country-wise association between other (non-traffic) injury and depression estimated by multivariable logistic regression. OR, Odds ratio; CI, Confidence interval. Models were adjusted for age, sex, wealth, education, setting (rural/urban), alcohol consumption and smoking. Overall estimates were obtained by meta-analysis with fixed effects.

The pooled OR (95% CI) for depression among individuals reporting a traffic injury was 1.72 (1.48–1.99) with a negligible level of between-country heterogeneity overall (*I*^2^ = 30.5%). However, there was a significant degree of heterogeneity between groups when analysed by country-income level (*p* = 0.043) with the pooled association strongest in UMICs (OR = 2.37) and weakest in LICs (OR = 1.46) (Fig. 3). Overall, other injuries were associated with 2.04 (95% CI = 1.85–2.24) times higher odds for depression (*I*^2^ = 29.6%) but in this case no significant between-group heterogeneity was observed (Fig. 4). In terms of any injury, the pooled OR (95% CI) was 1.96 (1.80–2.13) (*I*^2^ = 37.3%) and no significant heterogeneity by country-income was observed (online Supplementary Material Fig. S1).

## Discussion

This study used data from over 200 000 people in 40 LAMICs to examine the association between injury and depression. While the prevalence of each type of injury varied across individual countries a pooled estimate indicated that any past-year injury was common with 7.1% of the respondents across all countries experiencing some form of injury. Male sex, younger age, urban residence, alcohol consumption and smoking were all associated with recent injury. When plotting the prevalence of depression by the presence or absence of injury (Fig. 2), with just one exception (for traffic injuries in UMICs), experiencing injury was associated with a significantly higher likelihood of being depressed across all country income groups for both traffic and other forms of injury. This was particularly notable for other injuries where the prevalence of depression was doubled in LICs and LMICs and almost double in UMICs. Pooled estimates from fixed effects meta-analyses derived from cross-country multivariable logistic regression analyses showed that having a non-traffic (other) injury more than doubled the odds for reporting depression and that this effect was similar across different county groupings based on their level of wealth. Experiencing a traffic injury was associated with 1.72 times higher odds for depression but in this case, although there was only a negligible level of between-country heterogeneity, when analysed by country-income group the association was stronger in UMICs (OR: 2.37) and LMICs (OR: 1.73) compared to LICs (OR: 1.46).

This study supports calls for a greater priority to be given to injuries in LAMICs especially as both traffic-related and other injuries are common. The large variation in the prevalence of different types of injury between countries is, however, striking. There are many reasons why this might be so, both methodological, such as sampling and reporting differences, and contextual, such as traffic density, road safety and so on. Comparing these results with those from other studies is complicated by the fact that until now, there has been comparatively little research on injuries in LAMICs and those studies which have taken place have calculated the occurrence of injuries in different ways (Adeloye *et al*., 2016). Nonetheless, the findings from the current study in conjunction with those from earlier studies suggest that many people in LAMICs experience injury each year, but also, that the injury risk varies across countries and by the type of injury. This highlights the urgent need for more research on injuries in LAMICs so that the epidemiology of different forms of injury can be better understood in these countries.

The factors associated with injury are consistent with findings from other studies in LAMICs which have linked male sex, younger age and alcohol consumption to an increased risk for non-fatal injury/trauma (Peltzer *et al*., 2015; Taibo *et al*., 2016; Alonge *et al*., 2017). However, when specific forms of injury are considered the associations may be more nuanced. For example, a study using data from 14 LAMICs found different associations for injury due to road traffic accidents and falls by location (urban and rural areas) across low, lower middle- and upper middle-income countries (Raina *et al*., 2016). Similarly, other research has shown that alcohol consumption predisposes to different forms of injury (Taibo *et al*., 2016). Thus, while there might be certain general risk factors for injury, specific associations may depend on the particular population and/or type of injury being studied.

In pooled meta-analyses, there was a significant association between traffic and other (non-traffic) injuries and depression in all of the country income groups. This is consistent with results from several earlier studies that have linked injuries and depression/treatment for depression in LAMICs (Yiengprugsawan *et al*., 2012; Peltzer *et al*., 2015; Stewart Williams *et al*., 2015). A variety of factors might be important for the injury-depression association in LAMICs. It has been suggested for instance, that a failure to receive immediate or appropriate treatment may increase the risk of emotional distress (Peck, 2011). Injury can also result in disability, chronic pain and psychological stress (Stewart *et al*., 2016) which may lead to depression (Craig *et al*., 2009; Falla *et al*., 2016). Previous research has also highlighted that an injury can cause household financial hardship and food insecurity in LAMICs (Stewart *et al*., 2016), which other research has linked to common mental disorders (Lund *et al*., 2010).

For other injuries, the strength of the association was similar across all country income levels. However, for traffic injuries, there was a stronger association with depression in UMICs. It is uncertain what underlies this result and whether these factors might pertain to the form of traffic accidents, who is affected or the short/long-term consequences of traffic injuries in these countries. A previous study that used data from older Mexican adults (aged 50+) showed for example, that the percentage of those who received treatment for road traffic injuries was lower than in five other LAMICs, while the proportion that suffered physical disability was similar to or lower than in most of the countries (Peltzer *et al*., 2015). As an earlier report from the WHO also showed that middle-income countries bear a disproportionate burden of road traffic fatalities relative to their level of motorisation (World Health Organization, 2013), our finding highlights the need for more research to determine whether this association is observed in other UMICs, and if it is, to identify the factors that underlie it.

It is also important to acknowledge that the relationship between injury and depression is likely to be bidirectional (Patten *et al*., 2010) and that some people could have had depression prior to injury, with depression acting as a specific risk factor for injury. For example, an earlier study suggested that depression might increase the risk of injuries via a number of different mechanisms such as inattention, insomnia, or decreased vigilance (Peele and Tollerud, 2005). In particular, sleep disorders/problems, which have been described as one of the core symptoms of depression (Nutt *et al*., 2008) have themselves been linked to an increased risk for road traffic accidents (Moradi *et al*., 2018) as well as other types of injury such as work-related injuries (Uehli *et al*., 2014). It is also possible that depression is associated with other forms of behaviour that can result in physical injury such as self-harm/attempted suicide (Kessler *et al*., 1999; Mann, 2002; Skegg, 2005).

Before concluding, it should be noted that this study has several limitations. First, we lacked information about the specific form of the injuries, for example whether they were unintentional injuries, acts of self-injury, or suicide attempts, where they occurred, their severity and their sequelae. This absence of information reduces our ability to understand the observed association between injury and depression. For example, an earlier study from Thailand showed that the association between injury and depression may vary by location (transport, home, work, sport) (Yiengprugsawan *et al*., 2012). Second, major depressive episodes were not assessed with a clinical interview. However, this is common in large-scale epidemiological studies such as ours as this is often not logistically or financially possible. Third, it is possible that the higher odds we observed between other injuries and depression might have been a result of differences in the questions used. Specifically, the other injury question asked respondents if, their injury had ‘limited [their] everyday activities’ which may have indicated that these injuries might have been more severe. Fourth, it is possible that the occurrence of injury may have been underreported given that there is some evidence that injuries may be associated with an increased risk of experiencing social stigma in LAMICs (Stewart *et al*., 2016; Jagnoor *et al*., 2018). Fifth, it is also possible that we lacked information on potential confounders of the observed associations. In particular, we had no information on illicit drug use even though, for example, the use of cannabis may be linked to both an increased risk for depression (Degenhardt *et al*., 2003) and motor vehicle accidents and non-traffic injuries (Hall and Degenhardt, 2009; Barrio *et al*., 2012). Last, as this study used cross-sectional data it was not possible to establish causality or determine the direction of the associations.

While acknowledging that depression has a multi-faceted aetiology and that injury is just one possible causal factor, the finding that injury is strongly associated with depression in LAMICs has important implications given that depression has been associated with poorer post-injury outcomes (O'Donnell *et al*., 2005, 2009; Zatzick *et al*., 2008). It further reinforces the already strong case for giving greater priority to injury prevention in LAMICs (Wesson *et al*., 2014). This demands a comprehensive response, from strengthening and enforcement of road safety legislation (Staton *et al*., 2016) to improved trauma care (Wong *et al*., 2015), spanning treatment at the point of injury to rehabilitation (Reynolds *et al*., 2017). Unfortunately, as yet, there is little evidence on how to intervene for those suffering from the ongoing negative mental health effects of injuries in LAMICs, although a study from India reported that supportive psychotherapy may be associated with better psychological health among burns patients (Gouthi and Chadha, 2011).

This study raises almost as many questions as it answers. However, we hope that it will encourage a process in which those concerned with injuries and mental health can come together to develop coordinated solutions.

## Supplementary material

For supplementary material accompanying this paper visit http://dx.doi.org/10.1017/S2045796019000210. click here to view supplementary material

## Availability of data and materials

The World Health Survey is a publicly available dataset which is available for use upon request to the World Health Organization.
