# Supplementary material for: Injury and depression among 212 039 individuals in 40 low- and middle-income countries
Source: Epidemiol Psychiatr Sci. 2019 May 14;29:e32. doi: 10.1017/S2045796019000210 (PMC8063218; doi:10.1017/S2045796019000210)
Supplement: Supplementary file 1 [file S2045796019000210sup001.docx]

**Supplementary Material**

| **Table S1** Sample size and age- and sex- adjusted prevalence of traffic injury, other injury, any injury, and depression | | | | | | |
| --- | --- | --- | --- | --- | --- | --- |
| Country income | Country | n | Traffic injury | Other injury | Any injury | Depression |
| LIC | Bangladesh | 5,942 | 5.1 (4.3-5.9) | 5.3 (4.5-6.2) | 9.9 (8.9-11.1) | 11.5 (10.1-13.1) |
|  | Burkina Faso | 4,948 | 1.8 (1.4-2.3) | 4.3 (3.4-5.4) | 5.5 (4.5-6.6) | 7.7 (6.2-9.6) |
|  | Chad | 4,870 | 2.2 (1.6-3.0) | 3.8 (3.0-4.9) | 5.3 (4.2-6.5) | 11.3 (9.6-13.3) |
|  | Comoros | 1,836 | 2.3 (1.5-3.4) | 5.1 (3.8-6.8) | 6.5 (5.1-8.4) | 4.8 (3.7-6.3) |
|  | Ethiopia | 5,089 | 0.1 (0.1-0.3) | 1.6 (1.2-2.1) | 1.7 (1.2-2.3) | 7.4 (6.3-8.7) |
|  | Ghana | 4,165 | 3.1 (2.5-3.8) | 6.7 (5.8-7.8) | 9.1 (7.9-10.3) | 6.2 (5.2-7.5) |
|  | India | 10,687 | 3.5 (3.0-4.1) | 4.1 (3.3-5.0) | 7.2 (6.2-8.3) | 9.8 (8.6-11.2) |
|  | Ivory Coast | 3,251 | 3.5 (2.7-4.4) | 7.5 (6.1-9.1) | 9.8 (8.3-11.6) | 5.2 (4.0-6.8) |
|  | Kenya | 4,640 | 3.0 (1.9-4.7) | 12.1 (10.2-14.3) | 13.8 (12.1-15.8) | 9.0 (7.6-10.6) |
|  | Laos | 4,988 | 2.2 (1.8-2.8) | 1.7 (1.3-2.1) | 3.8 (3.2-4.4) | 1.3 (0.9-1.7) |
|  | Malawi | 5,551 | 3.1 (2.3-4.2) | 7.9 (6.7-9.2) | 10.7 (9.4-12.1) | 5.4 (4.6-6.3) |
|  | Mauritania | 3,902 | 1.7 (1.2-2.5) | 2.8 (2.0-3.8) | 3.9 (3.1-5.0) | 4.4 (3.3-5.8) |
|  | Myanmar | 6,045 | 0.9 (0.6-1.3) | 0.9 (0.5-1.6) | 1.7 (1.1-2.6) | 0.5 (0.3-0.8) |
|  | Nepal | 8,820 | 2.3 (1.9-2.8) | 3.3 (2.8-3.9) | 5.5 (4.8-6.3) | 9.6 (8.8-10.4) |
|  | Pakistan | 6,501 | 1.9 (1.4-2.5) | 1.9 (1.5-2.5) | 3.3 (2.7-4.1) | 6.5 (5.5-7.8) |
|  | Zambia | 4,165 | 1.4 (0.9-2.1) | 5.0 (4.1-6.1) | 6.2 (5.1-7.6) | 6.4 (5.3-7.7) |
|  | Zimbabwe | 4,290 | 1.7 (1.3-2.4) | 2.9 (2.3-3.7) | 4.3 (3.6-5.2) | 3.3 (2.5-4.4) |
|  | *Total* | *89,690* | *2.9 (2.6-3.2)* | *3.9 (3.6-4.2)* | *6.3 (5.9-6.8)* | *7.2 (6.8-7.7)* |
| LMIC | Bosnia Herzegovina | 1,031 | 2.1 (0.9-4.6) | 5.0 (3-8.4) | 6.7 (3.8-11.6) | 3.9 (2.6-5.6) |
|  | Brazil | 5,000 | 3.1 (2.6-3.7) | 7.9 (7.2-8.8) | 10.7 (9.8-11.6) | 15.8 (14.5-17.3) |
|  | China | 3,994 | 1.3 (0.8-2.2) | 1.5 (0.9-2.6) | 2.6 (1.8-3.7) | 0.8 (0.4-1.6) |
|  | Dominican Republic | 5,027 | 3.0 (2.4-3.7) | 4.2 (3.4-5.3) | 6.9 (5.9-8.1) | 7.4 (6.3-8.6) |
|  | Ecuador | 5,675 | 1.2 (0.8-1.8) | 4.3 (3.3-5.6) | 5.2 (4.1-6.6) | 5.5 (4.5-6.7) |
|  | Georgia | 2,950 | 0.8 (0.4-1.4) | 1.8 (1.2-2.6) | 2.4 (1.8-3.4) | 4.1 (3.1-5.5) |
|  | Kazakhstan | 4,499 | 1.3 (0.7-2.2) | 2.6 (1.9-3.6) | 3.3 (2.5-4.4) | 3.8 (2.8-5.2) |
|  | Morocco | 5,000 | 2.1 (1.5-2.9) | 3.7 (2.9-4.8) | 5.6 (4.6-6.8) | 18.9 (16.3-21.8) |
|  | Namibia | 4,379 | 2.5 (1.9-3.3) | 4.4 (3.4-5.6) | 5.9 (4.8-7.3) | 5.4 (4.4-6.7) |
|  | Paraguay | 5,288 | 1.5 (1.1-1.8) | 6.8 (6.0-7.7) | 8.0 (7.2-8.9) | 5.2 (4.5-6.0) |
|  | Philippines | 10,083 | 2.6 (2.2-3.0) | 3.4 (2.9-3.9) | 5.3 (4.7-6.0) | 2.6 (2.2-3.1) |
|  | Russia | 4,427 | 1.2 (0.8-1.8) | 8.6 (6.7-11.1) | 9.4 (7.4-12.0) | 3.6 (2.8-4.5) |
|  | South Africa | 2,629 | 3.7 (2.8-5.0) | 4.7 (3.6-6.0) | 7.1 (5.8-8.8) | 4.6 (3.4-6.2) |
|  | Tunisia | 5,202 | 1.7 (1.3-2.2) | 3.9 (3.2-4.7) | 5.4 (4.7-6.4) | 7.8 (6.6-9.2) |
|  | *Total* | *65,184* | *2.7 (2.4-3.0)* | *6.1 (5.7-6.6)* | *8.2 (7.7-8.7)* | *8.8 (8.3-9.4)* |
| UMIC | Croatia | 993 | 2.5 (1.4-4.4) | 6.4 (4.7-8.7) | 8.6 (6.5-11.3) | 4.0 (3.0-5.5) |
|  | Czech Republic | 949 | 3.0 (1.5-5.9) | 7.4 (4.9-10.9) | 10.1 (7.0-14.3) | 3.8 (2.6-5.5) |
|  | Estonia | 1,020 | 1.6 (0.9-2.9) | 6.6 (4.6-9.3) | 8.0 (5.7-11.0) | 6.0 (4.8-7.5) |
|  | Hungary | 1,419 | 2.5 (1.7-3.7) | 8.1 (6.4-10.2) | 10.0 (8.2-12.2) | 3.5 (2.7-4.7) |
|  | Latvia | 929 | 2.4 (1.2-4.8) | 10.8 (8.0-14.6) | 13.2 (10.1-17.1) | 4.9 (3.4-7.0) |
|  | Malaysia | 6,145 | 4.3 (3.7-5.0) | 4.4 (3.8-5.2) | 7.8 (7.0-8.7) | 1.3 (1.0-1.7) |
|  | Mauritius | 3,968 | 1.5 (1.1-2.1) | 6.0 (5.2-6.9) | 7.3 (6.4-8.3) | 7.4 (6.2-8.8) |
|  | Mexico | 38,746 | 1.8 (1.6-2.0) | 6.1 (5.7-6.6) | 7.7 (7.3-8.2) | 6.3 (5.9-6.8) |
|  | Uruguay | 2,996 | 1.4 (0.6-2.9) | 3.1 (2.3-4.2) | 4.2 (3.5-5.0) | 4.1 (3.4-5.0) |
|  | *Total* | *57,165* | *2.4 (2.2-2.6)* | *5.9 (5.5-6.2)* | *7.9 (7.5-8.3)* | *4.9 (4.6-5.2)* |

Abbreviation: LIC Low-income countries; LMIC Lower middle-income countries; UMIC Upper middle-income countries

All age-sex adjusted weighted estimates were calculated using the United Nations population pyramids for the year 2010.

**Figure S1** Country-wise association between any injury and depression estimated by multivariable logistic regression

Abbreviation: OR Odds ratio; CI Confidence interval

Models were adjusted for age, sex, wealth, education, setting (rural/urban), alcohol consumption, and smoking.

Overall estimates were obtained by meta-analysis with fixed effects.
